# Cardiovascular fitness associated with cognitive performance in heart failure patients enrolled in cardiac rehabilitation

**DOI:** 10.1186/1471-2261-13-29

**Published:** 2013-04-16

**Authors:** Sarah Garcia, Michael L Alosco, Mary Beth Spitznagel, Ronald Cohen, Naftali Raz, Lawrence Sweet, Richard Josephson, Joel Hughes, Jim Rosneck, Morgan L Oberle, John Gunstad

**Affiliations:** 1 Department of Psychology, Kent State University, Kent, OH, 44242, USA; 2 Summa Health System, Akron, OH, USA; 3 Brown University, Providence, RI, USA; 4 Wayne State University, Detroit, MI, USA; 5 Department of Medicine, Case Western Reserve University School of Medicine, Harrington Heart & Vascular Institute, University Hospitals Case Medical Center, Cleveland, OH, USA

**Keywords:** Heart failure, Cardiac rehabilitation, Metabolic equivalents task

## Abstract

**Background:**

Reduced cognitive function is common in persons with heart failure (HF). Cardiovascular fitness is a known contributor to cognitive function in many patient populations, but has only been linked to cognition based on estimates of fitness in HF. The current study examined the relationship between fitness as measured by metabolic equivalents (METs) from a standardized stress test and cognition in persons with HF, as well as the validity of office-based predictors of fitness in this population.

**Methods:**

Forty-one HF patients enrolled in cardiac rehabilitation completed a standardized exercise stress test protocol, a brief neuropsychological battery, the 2-minute step test (2MST), and a series of medical history and self-report questionnaires.

**Results:**

Maximum METs from stress testing demonstrated incremental predictive validity for attention (β = .41, *p* = .03), executive function (β = .37, *p* = .04), and memory domains (β = .46, *p* = .04). Partial correlations accounting for key medical and demographic characteristics revealed greater METs was associated with the 2MST (*r* (32) = .41, *p* = .02) but not with the Duke Activity Status Index (DASI) (*r*(32) = .24, *p* = .17).

**Conclusion:**

The current findings indicate that better fitness levels measured by METs is independently associated with better cognitive function in older adults with HF. Results also showed that METs was closely associated with one office-based measure of fitness (2MST), but not another (DASI). Prospective studies are needed to clarify the mechanisms linking fitness and cognitive function in HF.

## Background

Heart failure (HF) affects nearly six million Americans and is a leading cause of hospitalization and mortality in older adults [1,2]. Though less commonly examined than other consequences, cognitive impairment is also common in HF and believed to affect up to 75% of this population [3]. Deficits can be found in all cognitive domains, but are particularly pronounced on tests of executive function, attention, and memory [4,5]. Deficits in these cognitive domains can have adverse impact on patient outcomes, including reducing the ability to comply with medical regimen (e.g. medications, exercise, diet) and recognize symptoms of decreasing health [4].

Past studies have shown that reduced cerebral blood flow [6,7] and structural brain changes including greater global and region-specific atrophy [8,9], are important factors for the development of cognitive impairment in HF. Low physical fitness is another likely contributor to poor cognitive performance in persons with HF [10,11]. Only one previous study has examined the association between fitness and cognitive function in HF [12]. Findings were consistent with expectations, with poorer fitness levels being associated with greater cognitive impairment. However, that study utilized an office-based estimate of fitness, the 2 minute step test, rather than a more detailed assessment such as stress testing. Furthermore, past work has shown a strong relationship between cognitive dysfunction and decreased fitness in other populations, including chronic obstructive pulmonary disorder, [13] depression, [14] and healthy older adults [15].

Metabolic equivalents (METS) are often used to illustrate the intensity of tasks individuals are able to accomplish, providing a standardized definition of fitness. As such, METS are important constructs in understanding the fitness of HF patients and determining the types of activities they are able to do independently. Found to be valid estimates in other populations [16-18], both the Duke Activity Status Index (DASI) and the 2-minute step test are commonly used with HF patients to estimate METS. While these estimates have good clinical function, it has yet to be determined how well they estimate fitness in the HF population. Likewise, no study has yet examined the independent contribution of METS on cognitive function in HF patients.

Given these findings, the proposed study had two primary goals. First, we sought to clarify the possible association between fitness levels as measured by stress testing and cognitive function in persons with HF. Second, to examine the validity of office-based estimates of fitness by correlating them to performance on stress testing. Specifically, we examined the association between stress testing and the self report Duke Activity Status Index (DASI) and the objective 2-minute step test. Based on past literature in other populations, we hypothesized that metabolic equivalents (METs) would independently predict cognitive function in multiple domains. Based on their association with other measures of fitness, we also expected that office-based measures of fitness, specifically the 2-minute step test (2MST) and the Duke Activity Status Index (DASI), would be correlated to cognitive performance.

## Methods

### Participants

A sample of 41 older adults with HF was recruited from Summa Health System in Akron, OH. See Table 1. All participants were between 50-85 years of age, English-speaking, and had an established diagnosis of New York Heart Association class II or III at the time of enrollment. Exclusion criteria included a history of significant neurological disorder (e.g. dementia, stroke), head injury, severe psychiatric disorder (e.g. schizophrenia, bipolar disorder), substance use, and renal failure.

**Table 1:** Demographic and clinical characteristics of 41 older adults with heart failure

**Demographic characteristics**	
Age, mean (SD) years	68.34 (8.41)
Gender (% Women)	34.1
Race, (% Caucasian)	85.4
Years of Education, mean (SD)	13.59 (3.04)
**Clinical characteristics**	
LVEF (%)	39.00 (10.79)
Hypertension (% positive history)	68.3
Diabetes (% positive history)	34.1
BDI-II, mean (SD)	6.83(8.44)
**Physical fitness**	
METS, mean (SD)	6.24(311)
2MST, mean (SD)	69.56 (23.07)
DASI peak oxygen uptake (mL/min), mean (SD)	26.32 (6.17)

*Note*. *LVEF* Left Ventricular Ejection Fraction, *BDI-II* Beck Depression Inventory-II, *METS* Metabolic Equivalent Task; *2MST* 2-Minute Step Test; *DASI* Duke Activity Status Index.

### Instrumentation

#### Neuropsychological tests

A neuropsychological battery was conducted by trained research assistants under the supervision of a licensed clinical neuropsychologist. The following tests were selected as they are clinically useful and have been used to assess cognition in HF in previous studies [4,5]. The following cognitive domains were included:

##### Global cognitive function

The Modified Mini-Mental State Examination (3MS) is brief screening measure that provides an estimate of global cognitive function [19].

##### Attention

Attention was assessed using Letter Number Sequencing (LNS), the Trail Making Test A (TMT-A), and the Symbol Digit Modalities Test. The LNS asks participants to repeat unordered strings of letters and numbers in both numerical and alphabetical order. Higher scores reflect better executive functioning and this task has been shown to correlate with other measures (r = -.36) [20,21]. In the TMT-A participants are instructed to draw a connecting line between numbered circles as quickly as possible [19]. Completion time is a reliable and valid measure of complex visual scanning and psychomotor speed (Test-retest reliability estimated at r = 0.79) [22]. The Symbol Digit Modalities Test asks participants to code numbers to correspond with symbols as quickly as possible. Past studies have indicated this test is reliable and valid [23,24], with a test-retest reliability of r = 0.70 [25].

##### Executive function

To test executive function performance, the Frontal Assessment Battery, the Trail Making Test B (TMT-B), and the Stroop Interference Task were used. The Frontal Assessment Battery contains six subtests which assess various aspects of executive function, including conceptualization, mental flexibility, inhibition, and sequencing. Higher scores indicate better test performance [26]. The FAB has been shown to have strong psychometric properties including good internal consistency (Cronbach’s a = 0.78), discriminant validity (89.1% of cases were correctly identified), and interrater reliability (= 0.87, p < 0.001) [27]. The TMT-B requires participants to draw a connecting line between alternating numbered and lettered circles as quickly as possible [19]. Test completion time is a widely used measures of executive function, with strong psychometric properties (Test-retest reliability estimated at r = 0.89) [22]. The SCWT consists of three-45 second trials. The first involves reading as many words written in black ink as possible (Word). The next test requires participants to identify the ink color of blue, red, or green X’s (Color). The final test has words written in opposite color; for example, the word “red” is written in green ink and the person must response “green” (Color-Word). An interference score can be calculated, which anchors for overall talking speed and color perception while quantifying an individual’s ability to suppress prepotent response [28,29].

##### Memory

Memory tests included the California Verbal Learning Test-Second Edition (CVLT) and the Complex Figure Test (CFT). The CVLT asks participants to learn, recall, and recognize items from a 16-item word list. This test incorporates indices of delayed recall and recognition, and the average of these scores were used for analysis. This task has strong psychometric properties, including a high correlation to other verbal memory measures (r =0.63 to r = 0.96) [30]. In the CFT, participants recall a complex geometric design [31]. Interrater reliability and internal consistency reliability for this measure are strong in geriatric populations [32].

##### Language

To assess language performance, the Boston Naming Test-II (BNT-II) and the Animal Naming task were used. The BNT-II requires individuals to name pictures of objects ranging from high to low familiarity [19]. The BNT is closely correlated with other verbal ability tests such as the McGintie Reading Test (r = .83) [33]. Likewise, the Animal Naming task asks participants to generate as many animal names as possible within 60 seconds [34]. This task has been widely used as a measure of semantic verbal fluency in older adults [35].

#### Cardiovascular fitness level

Estimated metabolic equivalents (METs) were estimated using a standardized symptom limited volitional maximal treadmill stress test. In this test, treadmill elevation increased every 60 seconds and an increase in speed every 3 minutes as to estimate an increase in workload of 15%. The stress test ended if either the participant requested or if the participant showed clinical signs of cardiovascular, neurologic, or musculoskeletal distress. Maximum METs were estimated through a combination of treadmill speed, grade, and duration [36].

#### Office-based estimates of cardiovascular fitness

The two minute step test (2MST) requires individuals to walk in place, lifting their knees to a target midway between their kneecap and the crest of the iliac [37]. Individuals may use a wall or chair for balance as needed. A higher step count indicates better cardiovascular fitness [12].

The Duke Activity Status Index (DASI) was also administered to estimate individual's estimated peak oxygen uptake. This measure is a 12-question self-report on various aspects of physical work capacity and quality of life, asking whether individuals can complete daily tasks that are weighted based on the task's difficulty. It has been used as a valid estimate of physical ability in older adults [38].

#### Depressive symptoms

The Beck Depression Inventory II (BDI-II) was used to measure the presence and severity of depression [39]. This measure is a 21-item self-report instrument and has good psychometric properties including strong internal reliability and validity [40]. Higher scores on the BDI-II indicate more severe depressive symptoms.

### Procedure

All procedures were approved by the Kent State University and Summa Health Institutional Review Boards and written consent was obtained from all participants. METs were established with an exercise stress test protocol that was completed upon enrollment into cardiac rehabilitation. Within two weeks of the initial stress test, participants completed a research visit that consisted of a neuropsychological test battery, the 2MST, and series of medical history and self-report questionnaires, including the DASI. Ejection fraction was determined using medical record review.

### Statistical analyses

To facilitate clinical interpretation and to maintain directionality within the scales, all raw scores of the neuropsychological measures were transformed to T*-*scores (a distribution with a mean of 50, and a standard deviation of 10) using normative data correcting for age, similar to the approach used in other studies [41]. Memory measures were also corrected for gender. Composite scores for attention, executive function, memory, and language were means of the T*-*scores within each cognitive domain. Consistent with convention in many clinical settings, impairment in these domains for the current study was defined as a T-score ≤ 35 relative to normative data.

To examine the independent association between METs and cognitive function, a series of hierarchical regression analyses were performed with composite scores for each cognitive domain. All analyses utilized IBM SPSS Statistics version 21. For each model, age, sex (0 = female; 1 = male), years of education, LVEF, depressive symptomatology (as assessed by the BDI-II), and diagnostic history of hypertension and diabetes (1 = positive history; 0 = negative history), were entered into the first block. METs was then entered into the second block of each model to determine its incremental predictive validity above and beyond demographic and medical characteristics. Finally, a series of partial correlations adjusting for the aforementioned demographic and medical characteristics was conducted to examine the association of METs with the 2MST and peak oxygen uptake as estimated by the DASI.

## Results

### METs and cognitive performance

The current sample obtained an average maximum of 6.24 (SD = 3.11) METs during the stress test. See Table 1. On a global measure of cognitive function (3MS), average performance was 92.98 (SD = 5.36), with 22.0% of the sample scoring below 90, 41.5% between 90 and 95 and 36.6% above 95. See Table 2.

**Table 2:** **Neuropsychological test performance in older adults with HF (*N*** **= 41)**

	**Raw test performance, mean (SD)**	**T-score, mean (SD)**
*Global cognitive function*	92.98 (5.36)	**--**
*Attention*		
TMTA (seconds)	40.22 (14.47)	50.46 (8.65)
Letter number sequence	8.93(2.49)	51.37 (8.42)
Digit symbol	53.78 (11.78)	50.07 (7.88)
*Executive function*		
TMTB (seconds)	121.44 (77.24)	43.20 (22.44)
Frontal assessment battery	15.88 (2.46)	46.98 (20.78)
Stroop interference	1.04 (7.39)	51.05 (7.26)
*Memory*		
CVLT LDFR	7.85 (3.22)	46.95 (10.18)
CVLT total hits	13.59 (2.22)	44.88 (13.11)
CVLT false positive	4.46 (3.23)	48.66 (9.94)
CFT delayed recall	12.77 (6.13)	49.41 (8.82)
*Language*		
Boston naming test	53.61 (6.41)	51.38 (16.85)
Animals	19.93 (4.99)	56.27 (11.11)

*TMTA* Trail Making Test A; *TMTB* Trail Making Test B; *LNS* Letter Number Sequencing; *CVLT* California Verbal Learning Test; *LDFR* Long Delay Free Recall; *CFT* Complex Figure Test.

### METs is independently associated with cognitive function

Demographic and medical factors were associated with test performance for attention (*F*(7,33) = 2.39, *p* = .04) and executive function (*F*(7,33) = 2.91, *p* = .02). After controlling for age, sex, education, LVEF, depressive symptomatology (as assessed by the BDI-II), and history of hypertension and diabetes, METs demonstrated incremental predictive validity for attention (β = .41, *p* = .03), executive function (β = .37, *p* = .04), and memory domains (β = .46, *p* = .04). In each case, higher METs levels were associated with better cognitive test performance. No such pattern emerged for language abilities (β = .27, p = .23). Notably, bivariate correlations also revealed higher METs were associated with better performance on the 3MS (*r*(39) = .38, *p* = .01). See Table 3 for a full summary of hierarchical regression analyses.

**Table 3:** **METs independently predict cognitive function in older adults with heart failure (*N*** **= 41)**

	**Attention**	**Executive**	**Memory**	**Language**
	***b(SE b)***	***b(SE b)***	***b(SE b)***	***b(SE b)***
*Block 1*				
Age	.27(.14)	.65(.27)*	.25(.18)	-.13(.25)
Sex	2.51(2.47)	5.80(4.77)	.27(3.25)	4.38(4.50)
Education	.72(.38)	1.28(.73)	.36(.50)	1.07(.69)
LVEF	.00(.10)	.18(.10)	.03(.13)	-.05(.17)
BDI-II	.03(.14)	-.49(.27)	.08(.18)	-.32(.25)
Hypertension	−4.98(2.52)	−2.74(4.86)	−1.95(3.32)	−1.80(4.59)
Diabetes	−2.36(2.29)	−3.74(4.41)	−1.42(3.01)	−1.22(4.17)
*R*^*2*^	.34	.38	.11	.15
*F*	2.39*	2.91*	.58	.84
*Block 2*				
METs	.92(.41)*	1.67(.80)*	1.16(.54)*	.95(.78)
*R*^*2*^	.43	.46	.22	.19
*F* for ΔR^2^	5.10*	4.39*	4.57*	1.48

*Note.* *denotes p < 0.05; **denotes p < .01.

*Abbreviations*: *b* unstandardized regression coefficients; *SE* standard error; *LVEF* Left Ventricular Ejection Fraction; *BDI-II* Beck Depression Inventory-II; *METs* Metabolic Equivalent Task.

### Association between METs with the 2MST and DASI

To clarify the association between different estimates of fitness level, partial correlations accounting for the effect of medical and demographic characteristics revealed increased METs was associated with better 2MST performance (*r*(32) = .41, *p* = .02). No relationship emerged between METs estimated from stress test and peak oxygen uptake as estimated by the DASI (*r*(32) = .24, *p* = .17).

Consistent with this and the above pattern, partial correlations showed lower 2MST performance was also associated with worse attention (*r*(32) = .34, *p* = .047) and executive function (*r* (32) = .40, *p* = .02) even after accounting for age, sex, education, LVEF, depressive symptomatology, and diagnostic history of hypertension and diabetes. The 2MST was not associated with memory (*r*(32) = .13, *p* = .46) or language performance *(r*(32) = .15, *p* = .41)*.* Peak oxygen uptake as estimated by the DASI was not independently associated with cognitive function in any of the domains: attention (*r*(32) = .02, *p* = .90), executive function (*r*(32) = .05, *p* = .78), memory (*r*(32) = -.02, *p* = .91), language (*r*(32) = .17, *p* = .34).

## Discussion

Results from the current study indicate that a better fitness level as estimated by METs is associated with better cognitive function in older adults with HF. This relationship remained significant after controlling for key demographic and medical factors, including LVEF. A similar pattern emerged for one office-based estimate of fitness (i.e. 2MST) but not another (i.e. DASI). Several aspects of these findings warrant brief discussion.

Results showing that METs predicted cognitive function above and beyond ejection fraction are consistent with past work showing fitness is an important contribution to cognition in other healthy and medical populations [42,43]. There are several mechanisms by which better fitness levels may promote cognitive function in older adults with HF. A likely mechanism is increased cerebral blood flow (CBF). Greater CBF is associated with better cognition in HF [6,8] and greater CBF has also been associated with increased METs in other populations [44]. Similarly, better fitness levels may also be associated with better brain integrity in HF. For instance, lower physical activity levels have been associated with greater atrophy in white [45] and grey matter in older adults [46]. In turn, this and other forms of brain pathology have been linked to poorer cognitive function in persons with HF [47,48]. Future studies are needed to more clearly determine the mechanisms for the neuroprotective effect of fitness in HF patients, particularly prospective studies involving neuroimaging.

As noted above, HF patients frequently experience cognitive decline [3], which can lead to negative outcomes such as increased mortality and hospitalization, as well as a decrease in quality of life [1,2,49]. As fitness level is at least partly modifiable in HF patients, future work should further investigate the potential cognitive benefits of structured exercise in HF patients. Past work has shown that participation in a cardiac rehabilitation program was associated with cognitive improvements in a mixed sample of persons with cardiovascular disease [50], and it is possible that HF patients may show similar improvements. If so, structured exercise programs may protect against cognitive decline. Furthermore, cognitive impairment has been linked to hospitalization [2], and thus a decrease in cognitive deficits via exercise programs may decrease hospitalization rates and also ultimately reduce medical costs in HF.

We also examined the relationship between METs and two office-based measures of fitness to help clinicians identify those HF patients at risk for cognitive impairment. Found to be valid estimates in other populations [16-18], both the DASI and the 2MST have been suggested for use in HF patients. Contrary to expectations, although METs level was closely associated with 2MST, it was unrelated to the DASI in the current sample. The exact reason for the lack of association between METs and DASI is unclear, but may involve the cognitive impairment found in persons with HF. It is possible that HF-related cognitive impairment limits patients’ ability to provide accurate information about fitness levels. This is similar to work on the self report of sleep and functional status in which patients were unable to remember and make accurate judgments about their sleep length and quality as well as independent functioning [51,52]. As such, HF patients who suffer from executive dysfunction may be unable to accurately judge how much activity they engage in or how fit they truly are. Furthermore, participants may also have wanted to appear more functioning on the DASI than they may actually are, a common inaccuracy in self-report measures. Future work is needed to determine whether a similar amount of error is found in the report of fitness levels in persons with HF.

The current findings are limited in several ways. Studies are needed to clarify the mechanisms that link fitness levels and cognitive function in older adults with HF, particularly studies utilizing neuroimaging and direct measures of CBF. Future research would also benefit from prospective examination of fitness levels and cognitive function to better understand this relationship, particularly the possibility that programs like cardiac rehabilitation that may improve cognitive function in persons with HF. An additional limitation to the current work includes relatively small sample size and larger studies are needed to confirm the current findings.

## Conclusion

In summary, maximum METs is an independent predictor of cognitive function in HF patients. Future work is needed to clarify the underlying mechanisms for this relationship, particularly prospective studies to determine whether structured exercise programs may improve cognitive function in persons with HF.

## Abbreviations

HF: Heart failure; METs: Metabolic equivalents; 2MST: 2 minute step test; DASI: Duke activity status index; LVEF: Left ventricular ejection fraction; 3MS: Modified mini mental status exam; LNS: Letter number sequencing; TMT-A: Trail making test A; TMT-B: Trail making test B; CVLT: California verbal learning test; CFT: Complex figure test; BNT-II: Boston naming test-II; CBF: Cerebral blood flow

## Competing interests

The authors declare that they have no competing interests.

## Authors’ contributions

SG contributed to study concept and design, acquisition of subjects, data analysis and interpretation, and manuscript preparation. MA participated in study concept and design, acquisition of subjects, data analysis and interpretation, and manuscript preparation. MS, RC, NR, LS, LC, RJ, JH, JR, LO, and JG contributed to contributed to study concept and design, data analysis and interpretation, and manuscript preparation. All authors read and approved the final manuscript.

## Pre-publication history

The pre-publication history for this paper can be accessed here:

http://www.biomedcentral.com/1471-2261/13/29/prepub
